# Systematic analysis of transcribed loci in ENCODE regions using RACE sequencing reveals extensive transcription in the human genome

**DOI:** 10.1186/gb-2008-9-1-r3

**Published:** 2008-01-03

**Authors:** Jia Qian Wu, Jiang Du, Joel Rozowsky, Zhengdong Zhang, Alexander E Urban, Ghia Euskirchen, Sherman Weissman, Mark Gerstein, Michael Snyder

**Affiliations:** 1 Molecular, Cellular and Developmental Biology Department, KBT918, Yale University, 266 Whitney Avenue, New Haven, Connecticut 06511, USA; 2 Computer Science Department, Yale University, 51 Prospect St., New Haven, Connecticut 06511, USA; 3 Molecular Biophysics and Biochemistry Department, Yale University, 260 Whitney Avenue, New Haven, Connecticut 06511, USA; 4 Genetics Department, Yale University, 333 Cedar Street, New Haven, Connecticut 06511, USA

## Abstract

**Background:**

Recent studies of the mammalian transcriptome have revealed a large number of additional transcribed regions and extraordinary complexity in transcript diversity. However, there is still much uncertainty regarding precisely what portion of the genome is transcribed, the exact structures of these novel transcripts, and the levels of the transcripts produced.

**Results:**

We have interrogated the transcribed loci in 420 selected ENCyclopedia Of DNA Elements (ENCODE) regions using rapid amplification of cDNA ends (RACE) sequencing. We analyzed annotated known gene regions, but primarily we focused on novel transcriptionally active regions (TARs), which were previously identified by high-density oligonucleotide tiling arrays and on random regions that were not believed to be transcribed. We found RACE sequencing to be very sensitive and were able to detect low levels of transcripts in specific cell types that were not detectable by microarrays. We also observed many instances of sense-antisense transcripts; further analysis suggests that many of the antisense transcripts (but not all) may be artifacts generated from the reverse transcription reaction. Our results show that the majority of the novel TARs analyzed (60%) are connected to other novel TARs or known exons. Of previously unannotated random regions, 17% were shown to produce overlapping transcripts. Furthermore, it is estimated that 9% of the novel transcripts encode proteins.

**Conclusion:**

We conclude that RACE sequencing is an efficient, sensitive, and highly accurate method for characterization of the transcriptome of specific cell/tissue types. Using this method, it appears that much of the genome is represented in polyA+ RNA. Moreover, a fraction of the novel RNAs can encode protein and are likely to be functional.

## Abstract 2

RACE sequencing of ENCODE regions shows that much of the human genome is represented in poly(A)+ RNA.

## Background

Recent studies [1-5] have revealed that the composition and structure of the mammalian transcriptome is much more complex than was previously thought. Large-scale RT-PCR analysis to determine the structure of transcripts produced from exons of known human genes has shown that multiple transcripts are produced from most gene loci (an average of more than five was reported by Harrow and coworkers [6]). In many cases the 5' ends of these alternate transcripts are located more than 100 kilobases upstream from the previously known start site [1]. Likewise, systematic analysis of cloned mouse and human cDNAs revealed that many more transcripts than previously appreciated are transcribed from each known gene locus [7-9]. One source of complexity is alternative 5' ends; recent studies indicate that there are at least 36% more promoters than was previously recognized [10-14].

In addition to the diversity of transcripts from known loci, it appears that much more of the human genome is transcribed than was previously appreciated. Probing of tiling arrays with cDNA probes has indicated that there are at least twice as many transcribed regions of the human genome than had previously been annotated [3,15-18]. Rapid amplification of cDNA ends (RACE) analysis using primers designed to these novel transcribed regions (called transcriptionally active regions [TARs] or TransFrags) followed by hybridization to arrays confirms the transcription of these regions. However, this array analysis does not reveal information concerning transcript structure or abundance. The large number of these transcripts along with the fact that many long transcripts are produced suggest that much of the human genome is transcribed, at least at some level.

The different cDNA and tiling array studies to analyze transcription have also revealed extensive antisense transcription in mammalian genomes [2,19]. One concern is that these studies often use reverse transcription to create single-stranded cDNA, but this may also cause second strand synthesis. Thus, it is unclear whether the detected expression from the second strand is due to *bona fide *antisense transcription or a result of a probe made for the second strand.

These various studies have raised many more questions than have been answered. How much of the human genome produces transcripts that are present in the mRNA population? What is the nature of the transcripts produced by the novel transcribed regions? What fraction of novel transcribed regions is likely to be protein coding? What is the level of transcripts produced from the novel transcribed regions? Finally, how much antisense transcription occurs in human cells?

In an effort to address some of these questions and thereby better characterize the human genome and its gene annotation, we have systematically analyzed the transcribed loci in 420 selected portions of the ENCyclopedia Of DNA Elements (ENCODE) regions using 5'-RACE and 3'-RACE sequencing. The ENCODE regions are 44 regions that comprise 1% of the human genome and have been highly characterized with respect to transcripts and transcription factor binding [1]. Highly sensitive RACE sequencing provides new insight into the human genome and its transcription. We found that many genes not known to be expressed in a particular cell type produce properly spliced low abundance transcripts. We also found that in some cases the purported antisense transcription is likely to be an artifact of the reverse transcription reaction. Additionally, we systematically analyzed, for the first time, the structure and level of transcripts produced from many novel transcribed regions and from regions that were not known to be transcribed. RACE sequences derived from novel TARs showed that these regions are highly connected, and revealed the structure of several potential novel protein coding transcripts. Finally, we uncovered transcription in previous nontranscribed regions of the genome, demonstrating that much of the genome is transcribed. Overall, these studies significantly enhance our understanding of the transcriptome of the human genome.

## Results

### Overview of 5'-RACE and 5'-RACE sequencing experiments in selected ENCODE regions

We have studied the transcripts produced from annotated gene regions, novel TARs previously identified by high-density oligonucleotide tiling arrays, and regions that were not previously shown to be transcribed (nonTx regions) using 5'-RACE and 3'-RACE and DNA sequencing [15,18,20]. The chromosomal regions for our analysis are primarily from the ENCODE regions of chromosome 22, which is particularly well annotated, as well as additional ENCODE regions on chromosomes 11 and 21. The RNAs analyzed were from NB4 acute promyelocytic leukemia cells, HeLa cells, and placental tissue. Both polyA+ and total RNA were used. A summary of the experiments performed is presented in Table 1.

**Table 1:** Summary of RACE sequencing using polyA+ and total RNA from human cell lines and tissue

Experiment	Number of exon primers	Number of novel TAR primers	Number of nonTx primers	Number of sequence reads	Number of detected transcripts on the genome
1: NB4 total RNA	34	39	0	291	154
2: Hela polyA RNA	0	59	0	273	112
3: placenta total RNA	32	20	44	195	85
4: placenta polyA RNA	0	96	96	591	147

nonTx, region not previously shown to be transcribed; RACE, rapid amplification of cDNA ends; TAR, transcriptionally active region.

**Figure 1:**
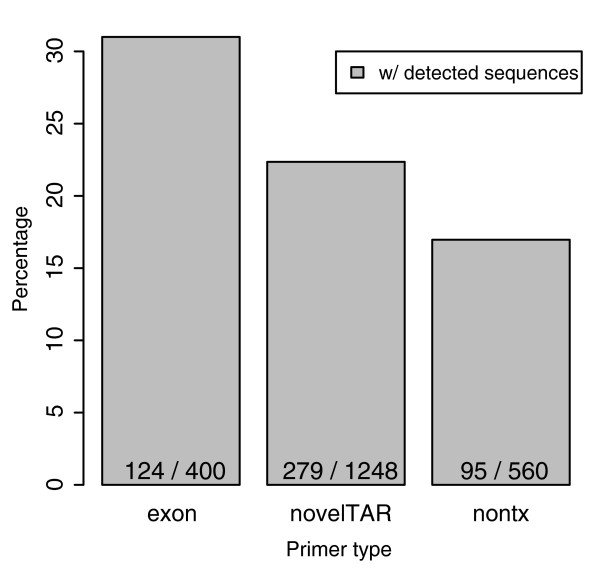
Frequency of PCR products obtained from different genomic regions. Primers designed to the sense and antisense strands of exons, novel transcriptionally active regions (TARs) and nontranscribed regions were used to generate rapid amplification of cDNA ends (RACE) products. The frequency of PCR products obtained is indicated. nontx, region not previously shown to be transcribed.

In total, 420 regions were analyzed; primers to each strand were designed and subjected to 5'-RACE and 3'-RACE reactions for a total of 1,680 reactions. Approximately 80% of the reactions generated products that were detected by gel electrophoresis (see Additional data file 1 for examples); 25% of these reactions yielded heterogeneous products (smears). The entire PCR reaction was subjected to DNA sequence analysis, and approximately 40% of the sequence reads mapped to the expected locations of the genome and were therefore deemed as products derived for the intended locus (see Materials and methods, below, for details regarding mapping of RACE sequences to the genome and the fitness score assignment). The average length of these sequence reads is 516 base pairs (bp). As expected, primers designed in known exons gave the highest proportion of valid RACE products. This is followed by the primers designed to the novel TARs. The nonTx regions gave the fewest RACE products (Figure 1). Similar results were observed with both polyA+ and total RNAs, as well as from human cell lines or tissue.

### RACE sequencing is highly sensitive in detecting transcripts expressed at a low level

**Figure 2:**
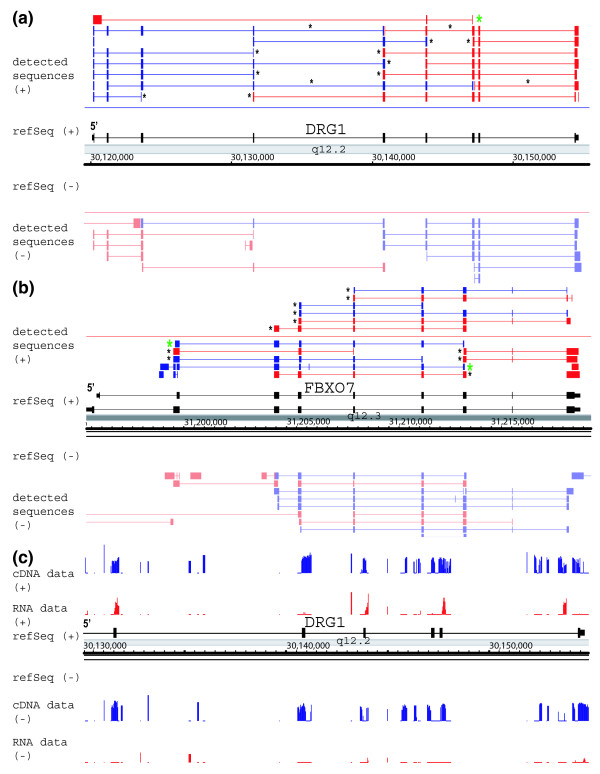
Distribution of RACE product sequences in the *DRG1 *and *FBX07 *regions. **(a) ***DRG1 *Region and **(b) ***FBX07 *region. Products from the sense strand (+) are shown in the top half of the panel. Products from the antisense strand are in the bottom half of the panel. Blue products are detected sequences from 5'-rapid amplification of cDNA ends (RACE); red products are detected sequences from 3'-RACE; black indicates refSeq; black asterisks indicate consensus splice sites (GT-AG, GC-AG, or AT-AC); and green asterisks indicate novel isoforms with more than 50% consensus splice sites. Note that the antisense products that lack consensus splice sites are indicated in lighter colors.**(c) **cDNA and RNA hybridization signals in DRG1 region. The blue tracks indicate the signals that were generated from hybridization of cDNA prepared from NB4 cells using reverse transcriptase to the strand-specific microarray. The red tracks indicate hybridization of RNA that has been labeled directly by chemical means, thus omitting the use of reverse transcriptase, to the strand-specific microarray. Products from the sense strand (+) are shown in the top half of the panel. Products from the antisense strand are in the bottom half of the panel.

We first analyzed the RACE sequences from eight known gene loci. For six of these loci we analyzed RNA from cells in which the gene was known to be expressed. For two genes, 5'-RACE and 3'-RACE reactions were performed using primers designed to the forward and reverse strand of each exon. For an additional four genes we analyzed a subset (1 to 8) of exons in the gene. As shown in Figure 2, the sequences of the known loci mostly matched the known annotations. For example, analysis of the *DRG1 *and *FBXO7 *genes, which are known to be expressed in NB4 cells, revealed cDNA sequences that matched the expected transcripts described in Refseq. In addition to detecting known transcripts, we also found novel isoforms. Some of these isoforms contained new exons whereas others contained different combinations of the known exons. An example is shown in Figure 2b for *FBXO7*. A novel exon was found for one of the RACE products and a novel combination was observed for another product. For the six genes analyzed we found evidence for 16 novel isoforms.

**Figure 3:**
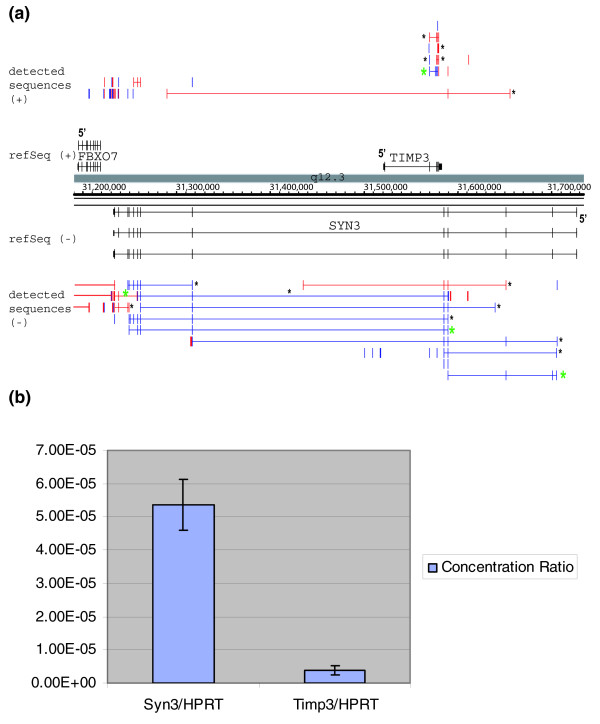
RACE sequencing can detect transcripts not previously detected by microarray analysis in NB4 cells. **(a) **Integrated Genome Browser (IGB) view *SYN3 *and *TIMP3 *rapid amplification of cDNA ends (RACE) products in NB4 RNA. **(b) **Real-time PCR quantification of *SYN3 *and *TIMP3 *transcripts relative to *HPRT1 *in NB4 cells.

We also analyzed expression of two gene loci, namely *SYN3 *and *TIMP3*, in cells in which their expression was not detected by tiling microarray analysis. *SYN3 *and *TIMP3 *are encoded on opposite strands from one another on chromosome 22. *SYN3 *(*Homo sapiens *synapsin III mRNA) encodes a neuronal phosphoprotein that is involved in synaptogenesis and in the modulation of neurotransmitter release, and it is implicated in several neuropsychiatric diseases such as schizophrenia [21,22]. *TIMP3 *encodes tissue inhibitor of metalloproteinase 3. Mutations in this gene have been associated with the autosomal dominant disorder Sorsby's fundus dystrophy [23]. NB4 RNA hybridization to high-density oligonucleotide tiling arrays did not produce signal above background in the *SYN3*/*TIMP3 *region. With RACE sequencing a number of products were observed. Most RACE sequences (eight) matched that of the annotated RefSeq isoforms for *SYN3 *(NM_003490.2). RACE sequences also revealed three other novel isoforms with exon skipping and intron inclusion (Figure 3a). Similar results were found for *TIMP3*. The presence of additional RNA isoforms suggests that additional messages are probably produced from each gene locus.

To gain a better understanding of why the *SYN3 *and *TIMP3 *genes were not detected by microarray analysis, we examined their expression level by real-time quantitative PCR. As shown in Figure 3b, the expression levels of *SYN3 *and *TIMP3 *are 1 × 10^4 ^and 1 × 10^5 ^times lower than that of the *HPRT1 *transcript. *HPRT1 *is expressed at low levels in various cell lines and tissue types, with fewer than 8 to 15 serial analysis of gene expression (SAGE) tags per 200,000 (<10^-5^), according to the SAGE Anatomic Viewer [24]. Thus, the transcripts produced by Syn3 and TIMP3 in NB4 cells are present at an extremely low level.

The novel RNA isoforms from annotated genes were examined for their ability to produce novel protein isoforms. The 16 novel RNAs identified in this study can produce five novel protein isoforms.

### A number of antisense transcripts detected in multiple regions appear to be artifacts

Antisense transcription plays diverse and important biologic roles, and recent studies using reverse transcription based approaches have reported a large amount of antisense transcription in the human genome [16,19]. Our study employed primers to analyze transcription from both DNA strands and thus examined antisense transcription. In addition to detecting transcription from the expected DNA strand, the RACE experiments produced sequences from the complementary strand for five of eight known gene loci. These sequences were revealed in experiments using both natural tissue and cell lines. However, careful inspection revealed that in most cases (29 out of 35) the splice junctions of most of the antisense products are not consistent with the GT-AG, GC-AG, or AT-AC pattern. Instead, they merely mirror (reverse complement) the splice junctions of the sense products. Two examples are shown in Figure 2 for the *DRG1 *region and the *FBX07 *regions (known genes on the plus strand). In these regions large numbers of antisense products (14 and 21, respectively) were detected on the opposite strand from both 5'-RACE and 3'-RACE reactions. Most of the antisense products lack the (GT-AG, GC-AG, or AT-AC) consensus splice sequences. It therefore appears likely that many of these antisense products are derived from the *in vitro *reverse transcription reaction, where double strand cDNAs might have formed [25], or from a complementary RNA *in vivo *[16].

To investigate further whether the antisnese transcript may be an artifact due to reverse transcription, we employed a novel strategy, namely direct chemical labeling of RNA followed by strand-specific oligonucleotide tiling microarray analysis. As shown in Figure 2c for the *DRG1 *locus, hybridization of cDNA prepared from NB4 cells using reverse transcriptase to the strand-specific microarray produced both sense and antisense signals. However, hybridization of RNA that has been labeled directly by chemical means, thus omitting the use of reverse transcriptase, usually yielded signals only from the annotated strand. This experiment indicates the much (but not all) antisense signal is directly tied to the use of reverse transcriptase and not likely to be present *in vivo*.

### Novel transcripts and their connectivity

**Figure 4:**
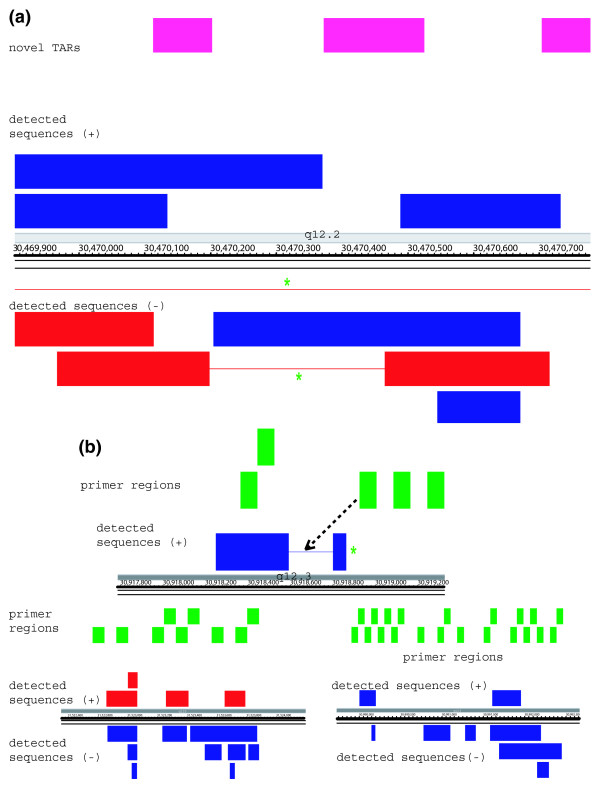
RACE products from novel TARs and nonTx regions. **(a) **novel transcriptionally active regions (TARs) and **(b) **regions not previously shown to be transcribed (nonTx regions). Pink indicates novel TARs, and green nonTx regions that the primers were designed from. Note that the products are primarily unspliced.

In addition to examining annotated genome regions, we analyzed a large number of novel TARs by RACE sequencing in order to gain a better understanding of their structure, their connectivity to known genes, and whether they might encode proteins of significant length. In all, 856 RACE reactions were generated to 214 TARs of the ENCODE regions [18]. End sequencing of the 5'-RACE and 3'-RACE products on both strands of the genome revealed overlapping sense and antisense transcripts (Figure 4a). This is consistent with recent work by Kapronov and coworkers [5,11,26] using RACE microarray experiments, although they did not analyze the transcript structure. In addition to analyzing TARs, we designed primers to 140 regions not known to produce transcripts; 17% of the primers were able to generate a RACE product whose sequence mapped to the expected region (Figure 1). Control experiments lacking reverse transcriptase do not produce products, indicating that the products are derived from RNA and not contaminating DNA. This frequency of successful RACE products from nonTX regions is lower than in known exon or novel TAR regions, but it nonetheless indicates that a substantial fraction of the human genome produces RNA. The transcripts from the nonTx regions exhibited an interleaved distribution similar to those from the novel TARs (Figure 4b).

**Figure 5:**
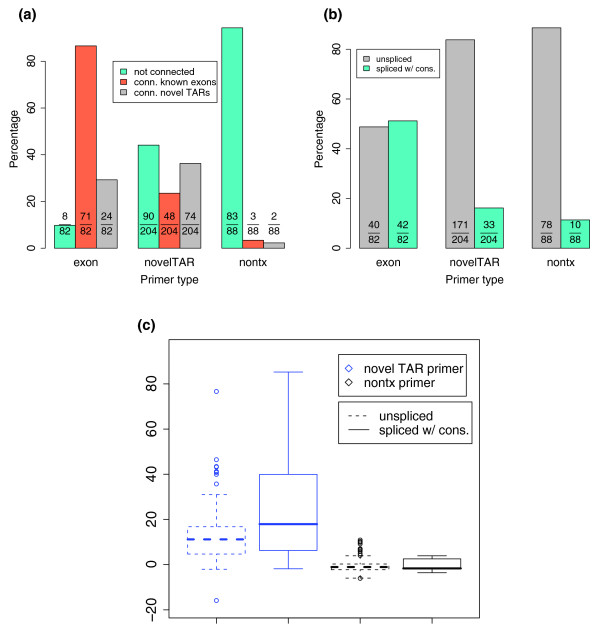
Features of the RACE products. **(a) **Connectivity of detected transcripts to known exons/novel transcriptionally active regions (TARs). **(b) **Frequency of splice and unspliced rapid amplification of cDNA ends (RACE) products derived from known exons, novel TARs, and untranscribed regions. **(c) **Average microarray intensities of regions encoding spliced and unspliced RACE products. nontx, region not previously shown to be transcribed.

The majority (85%) of the RACE sequences from the TARs and nonTX regions map contiguously (without introns) to the genomic sequence. Products from primers that lie close together on the genome often overlap one another or known exons, suggesting extensive transcription throughout the entire region. In addition, whereas the RACE sequences derived from known exons are mostly connected with known exons, the sequences from nonTx regions are rarely connected to others (Figure 5a). Although some of the regions yield results consistent with discrete transcripts, many do not.

Approximately 16% and 11% of the products produced from TARs and nonTx regions, respectively, produce transcripts that are spliced with consensus GT-AG, GC-AG, or AT-AC splice sequences (see Consensus splice site analyses [under Materials and methods, below]; Figure 5b). This is in contrast to products produced from exons in which approximately 50% of the messages are spliced. Moreover, further analysis of the novel TARs revealed that the RNA sequences with consensus splice sites originated from regions with higher microarray signal intensity on average than the unspliced ones (Figure 5c). Microarray signal intensity of the nonTx regions is close to background for both spliced and unspliced RACE sequences.

### Several newly transcribed regions are likely to produce protein

In order to determine better whether the novel transcripts may be functional, we examined their ability to encode protein. The sequences of RACE products were analyzed with respect to whether they contain open reading frames (ORFs) and/or whether the potential protein coding sequences are homologous to those in the nonredundant protein database. For two spliced sequences and 25 unspliced sequences, potential ORFs were found that have at least 50 codons, and the predicted protein sequence was homologous to that of a known protein present in the nonredundant database with a BLASTX threshold score of 1 × e^-9 ^[27]. The 27 transcripts contain 20 unique proteins, and nine out of 20 protein encoding ORFs have a translational start and stop codon (11 of the 27 transcripts).

**Figure 6:**
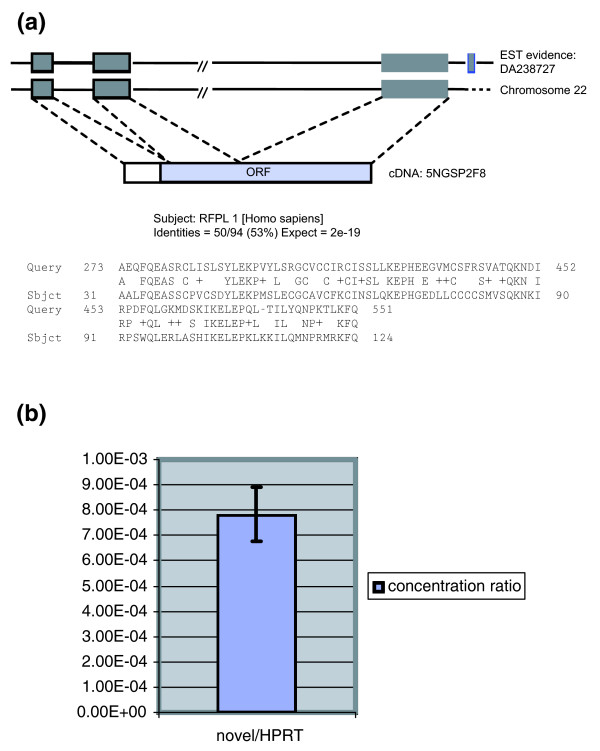
Example of a novel transcript detected by RACE sequencing. **(a) **Novel transcript 5NGSP2F8 (with consensus splice site) has a potential open reading frame of 142 amino acids; also, there is spliced expressed sequence tag (EST) evidence for it. **(b) **Real-time PCR relative quantification of the novel transcript to *HPRT1 *in placenta polyA+ RNA. RACE, rapid amplification of cDNA ends.

One example of a potential protein coding transcript is shown in Figure 6. The novel transcript 5NGSP2F8 detected by RACE end sequencing was properly spliced with a consensus pattern. It encodes a potential ORF that is 142 codons in length. Evidence for the transcript is also supported by a spliced expressed sequence tag (EST), although for 5NGSP2F8 the EST sequence contains a shorter ORF, presumably through DNA sequencing errors.

We examined the expression level of novel transcript 5NGSP2F8 using real-time quantitative PCR. The 5NGSP2F8 expression level is more than 1,000-fold lower than that of the *HPRT1 *transcript, indicating that the gene is expressed at a low level (Figure 6b).

## Discussion

Even though it is estimated that only 20,000 to 25,000 protein coding genes exist in the human genome, the transcriptome is quite complex and contains protein coding, nonprotein coding, alternatively spliced, and antisense genes [28]. RACE sequencing has provided a sensitive means for probing the human transcriptome. We found that transcripts from known gene regions often matched the known gene annotation but that many additional novel transcripts were also detected. We were also able to detect both novel and known RNA transcripts from known genes that were not previously detected in NB4 cells using genomic tiling arrays. It is thus likely that many (and possibly the majority) of known genes are expressed and spliced in human tissues and cell lines, and that multiple transcripts are produced from most gene loci, at least at a low level.

In addition to many annotated exons, high-density oligonucleotide tiling arrays has identified a large number (8,958) of novel TARs located in both intronic regions and intergenic regions distal from previously annotated genes [15,18]. In this report, end sequencing of the 5'-RACE and 3'-RACE PCR products from novel TARs identified extensively overlapping and interconnected novel transcripts. Most of the RACE sequences from the novel TARs and the nonTX regions are unspliced. This is consistent with mouse transcriptome studies, which found the most obvious difference between coding and noncoding transcripts to be that a higher percentage (71%) of the noncoding transcripts are unspliced/single exons, as compared with protein coding transcripts (18%) [29]. Many human RACE products do not contain long ORFs, and thus the function of these transcripts is not known. They probably either represent nonprotein coding RNAs that may have structural, enzymatic, or regulatory functions; pre-mRNAs; or RNAs from genomic regions that are transcribed and present in polyA+ RNA but lack a function.

Although many of the novel RNAs do not have long ORFs, a subset of them do (about 9%). From our limited study we found 27 protein coding sequences that are not present in RefSeq but are likely to encode proteins based on the presence of a more than 50-codon ORF that is homologous to other proteins in GenBank. A small fraction (two out of 27) of these is spliced. Additional studies of the entire human genome are thus likely to expand the number of protein coding genes accordingly.

Complementary natural antisense transcripts exert control at many steps of gene expression in prokaryotes and higher eukaryotes from transcription to translation, including transcript initiation, elongation, mRNA processing, location, and stability [30,31]. Natural antisense transcripts may be involved in diverse biologic functions, such as development, adaptive response, viral infection, and genomic imprinting [32,33]. In recent years, a large amount of sense-antisense transcription phenomena have been reported in both human and mouse. In a mouse transcriptome study using the reverse transcribed cDNA libraries [19], it was indicated that as many as 72% of all transcriptional units have an antisense transcript. In humans, 61% of all transcribed regions were suggested to possess antisense transcript [16]. Our findings that some antisense transcripts lack consensus splice junctions and can be detected on strand-specific microarrays only in cDNA, but not directly labeled RNA, raises the possibility that many antisense signals are artifacts resulting from reverse transcription. The conditions that we used are similar to those used by most other laboratories, suggesting that low level second strand synthesis is likely to be present in many studies. Consistent with this, while our manuscript was under review, Perocchi and coworkers recently reported the presence of *in vitro *antisense synthesis in their cDNA preparations [25]. These findings indicate that much antisense transcription is due to *in vitro *synthesis and not *in vivo *cDNA synthesis, and therefore caution should be used in interpreting antisense messages. The fact that some antisense regions still hybridize to directly labeled RNA probes indicates that some antisense transcripts do exist *in vivo*.

RACE sequencing was able to uncover novel transcripts from nontranscribed regions where microarray experiments did not detect any transcription, indicating the RACE sequence is more sensitive. This is probably due to the fact that micorarray signals are dampened by cross-hybridization to short oligonucleotides on the array. This problem is especially acute for genes that have homologous pseudogenes and paralogs. RACE sequencing offers several other advantages relative to microarrays. Microarrays do not provide information about transcript structure, splicing patterns, or the ability of these regions to encode proteins. Only sequencing full-length cDNA can resolve these issues. The recent developments of massively parallel sequencing technology has the potential to expedite this process greatly [34-37]. A large number of sequences (400,000 250-bp reads for 454 sequencer [Roche Applied Science, Indianapolis, IN, USA] and >300 million approximately 30-bp reads for Solexa sequencer [Illumina Inc., San Diego, CA, USA]) can readily be obtained in a single run. Although still relative short, these reads have the potential to identify novel transcribed regions of the human genome, and the longer reads may help to identify new spliced variants [38].

As noted above, quantitative measurements of transcript expression reveals that two known genes (*SYN3 *and *TIMP3*) are expressed at low levels even in tissues where they have no obvious role and cannot be detected by standard methods. Likewise, analysis of novel TARs and even random regions of the genome indicates that much of the genome produces transcripts that are present in polyA+ RNA, at least at a low level. Expression of these RNAs was 10^3 ^to 10^5 ^times lower than that of the *HPRT *gene. Assuming that *HPRT *is present at 10^-5 ^(1 copy per 100,000 molecules of the total RNA) in total RNA, the novel transcripts we detected are present at 10^-8 ^to 10^-10 ^of the total RNA. The finding that much of the genome is likely to be expressed has previously been reported for yeast, for which evidence also exists that the RNA is translated [39,40]. As suggested previously, we speculate that the ability to express novel regions of the genome continuously could ultimately be useful in evolution for selecting new functions.

Our study highlights the enormous complexity of the human transcriptome and the vast amount of RNA transcripts generated both from alternative splicing and protein coding and nonprotein coding RNAs. The ability of RNA to encode protein and to serve a structural and regulatory role makes it a diverse molecule for mediating many functions. The remarkable complexity of RNAs of the human transcriptome coupled with their diverse functions may therefore help explain the dramatic increase of complexity in higher eukaryotes and phenotypic variation [41,42].

## Materials and methods

### Target selection

The regions of our analysis are selected mainly from the chromosome 22 ENCODE region, with additional targets in chromosome 11 and 21 ENCODE regions. Except for a few regions for test purposes, we selected most of the exon and novel TAR primer regions from among those expressed (cell type specific) regions in known exons and novel TAR regions detected by transcriptional tiling array experiments. The nontranscribed primer regions are selected in a tiled manner from among those regions that are neither known exons nor novel TARs.

### Primer design

We designed four primers for each targeted region, which can be exons of known gene, TAR, or previously identified untranscribed regions. Two gene-specific primers (GSP1 and GSP2) and two nested GSPs (NGSP1 and NGSP2) on both plus and minus strand were selected for each targeted region using a modified Primer3 program. The primers are 23 to 28 nucleotides long, with GC content of 50% to 70% and with Tm (melting temperature) above 70°C (optimally 73°C to 74°C). Self-complementary primers that could form hairpin were avoided. We also voided complementarity between GSPs and UPM (universal primer A in the SMART RACE™ kit [Clontech, Mountain View, CA, USA]), particularly in their 3' Ends (UPM long: 5'-CTAATACGACTCACTATAGGGCAAGCAGTGGTATCAACGCAGAGT-3'; UPM short: 5'-CTAATACGACTCACTATAGGGC-3'). Complementarity between NGSPs and NUP (nested universal primer A), particularly in their 3' ends, was avoided (NUP: 5'-AAGCAGTGGTATCAACGCAGAGT-3'). The primers were mapped against the genome to ensure that they mapped to only one location (with identity <80% to other locations).

### 5'-RACE and 3'-RACE experiments, and end sequencing

Human NB4 cell line total RNA, Hela S3 polyA+ RNA, placenta total RNA, and polyA+ RNA (Ambion, Austin, TX, USA) were used in cDNA amplification by SMART RACE™ kit (Clontech), in accordance with the manufacturer's instructions [43]. 5'-RACE-Ready cDNA and 3'-RACE-Ready cDNA were synthesized using PowerScript (Clontech, CA, USA) or Superscript II (Invitrogen, CA, Carlsbad, USA) moloney murine leukemia virus reverse transcriptase and SMARTII A oligo (5'-AAGCAGTGGTATCAACGCAGAGTACGCGGG-3'), 5'-CDS primer A ([5'-(T)25V N-3' (N = A, C, G, or T; V = A, G, or C)]), or 3'-CDS primer A (5'-AAGCAGTGGTATCAACGCAGAGTAC[T]30V N-3' [N = A, C, G, or T; V = A, G, or C]). A total of 1 μg RNA was used in a final volume of 10 μl reverse transcription reaction (100 ng/ul). A reverse transcription reaction without reverse transcriptase was used as negative control to distinguish genomic DNA contamination. RACE was followed by PCR amplification using UPM and GSP1 or GSP2 on both strands of the genome. A 0.5 μl reverse transcription reaction from the above was used in 50 μl of PCR reaction in the Advantage™ 2 PCR Enzyme System (Clontech). Nested PCRs were performed using NUP (5'-AAGCAGTGGTATCAACGCAGAGT-3') and NGSP1 or NGSP2. One microliter of RACE PCR product was used in a 50 μl reaction. The PCR program was 94°C for 30 seconds and 72°C for 3 minutes, five cycles; then 94°C for 30 seconds, 70°C for 30 seconds and 72°C for 3 minutes, five cycles; followed by 25 cycles of 94°C for 30 seconds and 68°C for 30 seconds; and concluding with by an extension cycle of 72°C for 3 minutes. Nested PCR products were end sequenced using NGSP1 or NGSP2. The RACE sequences have been submitted to GenBank EST database (accession numbers from EW712308 to EW712635).

### Mapping RACE sequence to the genome

We first use the BLAT alignment tool [44] to compare all the RACE sequence reads to the human genome assembly (hg17, May 2004), and then evaluated the 'fitness scores' of the BLAT output matches using the following formulas:

*sizeDif *= abs([*tEnd *- *tStart*] - [*qEnd *- *qStart*]) + abs(*qSize *- [*qEnd *- *qStart*])

*insertFactor *= *qNumInsert *+ *tNumInsert*

*total *= *matches *+ *repMatches *+ *misMatches*

*badness *= (1,000 × *misMatches *+ *insertFactor *+ 3 × log[1 + *sizeDif*])/*total*

*fitness *= 100 - *badness *× 0.1

Where parameters such as *tEnd *have the same meanings as those defined in the BLAT documentation. The fitness score is based on the 'percent identity score' in the University of California at Santa Cruz Genome Browser [45], and it includes additional penalty on small overall matches. Once these fitness scores have been computed for one RACE experiment, a distribution of these scores was derived based on the characteristics of those BLAT matches that are located on the 'correct' chromosomes, and only those 'correct' matches with scores above a certain threshold were kept as 'valid' products and correspondingly as 'valid' transcripts. See Additional data file 2 for further details.

### Consensus splice site analyses

For those BLAT matches with multiple blocks, the corresponding splice sites in the transcripts were further examined in the following way. A splice site is defined as a consensus one if and only if a 'GT-AG' (or 'GC-AG'/'AT-AC', which appear much less often) pattern can be observed within windows of eight nucleotides on the two ends of it. (For example, for a splice site starting at chromosome position *i *and ending at *j*, the windows are [*i *- 3, *i *+ 5) and [*j *- 5, *j *+ 3].) An overall consensus score was then assigned to each transcript according the proportion of consensus splice sites in all its splicing events. We also used this consensus splice site criteria to filter out mirroring antisense transcripts caused by experimental artifacts. See Additional data files 3 for discussions on the choice of window size and the resulting transcripts after this filtering.

### Analyzing the correlation of signal intensity and transcript characteristics

Normalized signal intensities from across tiling array experiments were extracted for those primer regions and correspondingly assigned to the transcripts. These signal intensities were correlated with different transcript characteristics such as splicing events in our analysis.

### Analyzing the connectivity to known exons/novel TARs

The 'valid' transcripts were also compared against RefSeq gene annotation [46] and the union of all of the novel TARs for connectivity information. For each transcript, its connectivity to known RefSeq genes is the number of unique known exons that overlap with this transcript, and the connectivity to novel TARs is the number of novel TARs that overlap with this transcript.

#### Protein homology analysis of the novel transcripts

We consider a RACE sequence (either a single block one or with consensus splice sites) a 'novel transcript' if it is not connected to any RefSeq genes. We consider it a 'novel isoform' of a known gene if it overlaps with a known gene and has at least 50 bp not covered by existing annotation. We then compared all such novel transcripts to the nonredundant database using BLASTX [27], and selected those transcripts that have at least 50 amino acids significant matches in the database as candidates for further analysis. For the 'novel isoform' sequences, we then examined whether they encode a different protein domain.

#### Real-time RT-PCR

Human NB4 cell line total RNA or placenta polyA+ RNA (Ambion) were used to make 5'-RACE-Ready cDNA, as described above. Real-time quantitative PCR experiments were performed in quadruplication using LightCycler^® ^480 Probe Master or TaqMan^® ^Universal PCR Master Mix according to the manufacturer's instructions on a LightCycler^® ^480 system (Roche Applied Science, Indianapolis, IN, USA). Human *HPRT1 *endogenous control, human *SYN3 *(Hs00185853_m1), and *TIMP3 *(Hs00165949_m1) TaqMan^® ^gene expression assays were ordered from ABI (Applied Biosystems, Foster City, CA, USA). Real-time quantitative PCR primers for novel TAR RACE product 5NGSP2F8 (left primer: tacagcgcagccagaatg; right primer: gggcaggcaaagtcagataa; ProbeLibrary probe: #87; catalog no. 04689127001) were designed using Universal ProbeLibrary Assay Design Center [47] (Roche Applied Science). Primers were designed across a splice junction. The amplicon is 60 bp. Serial dilutions of cDNA template (400 ng, 100 ng, 25 ng, and 6.25 ng) were used in 20 μl real-time quantitative PCRs. The PCR program parameter was 50°C for 2 minutes and 95°C for 10 minutes, followed by 45 cycles of 95°C for 15 seconds and 60°C for 1 minute, and a final cooling step of 40°C for 10 seconds. The PCR amplification efficiencies among *HPRT1 *and our target transcripts are close (within 10%), and *HPRT1 *amplification is in the same linear range as our target transcripts. Roche relative quantification software was used to compare the relative expression levels of our target transcripts with *HPRT1*.

#### Direct labeling of total RNA and cDNA and hybridization to ENCODE tiling arrays

Total RNA and cDNA from human NB4 cells was chemically labelled with biotin using ULS reagent from Kreatech (Amsterdam, The Netherlands) for total RNA and LabelIT reagent from Mirus Bio (Madison, WI, USA) for cDNA. Five micrograms of total RNA and cDNA per array hybridization was incubated with labeling reagent for 30 minutes at 85°C and 60 minutes at 37°C, respectively. Samples were then purified with Qiagen PCR purification columns (Qiagen, Valencia, CA, USA) and ethanol precipitation, respectively. Labelled samples were hybridized to Affymetrix (Santa Clara, CA, USA) ENCODE 1.0 oligonucleotide tiling microarrays. Each sample was hybridized in triplicate to both the forward-strand and reverse-strand version of the array, using the manufacturer's standard hybridization, staining, and washing protocols. The arrays were scanned on an Affymetrix 7G Plus GeneChip scanner, and the signal intensity data were processed using a sliding window of 101 bp.

## Abbreviations

bp, base pairs; ENCODE, ENCyclopedia Of DNA Elements; EST, expressed sequence tag; GSP, gene-specific primer; NGSP, nested gene-specific primer; nonTx region, region not previously shown to be transcribed; ORF, open reading frame; RACE, rapid amplification of cDNA ends; RT-PCR, reverse transcription polymerase chain reaction; SAGE, serial analysis of gene expression; TAR, transcriptionally active region; UPM, universal primer A.

## Authors' contributions

Experiments were designed by JQW with suggestions from MS. Experiments were performed by JQW. Bioinformatics analyses were performed by JD; JR and ZZ helped with data analyses. AEU and GE contributed to direct labelling of total RNA. Experiments were performed in the laboratory of MS and SW. Bioinformatics analyses were performed in the laboratory of MG. All authors read and approved the final manuscript.

## Additional data files

The following additional data are available with the online version of this paper. Additional data file 1 shows examples of RACE PCR products on an agarose gel. Additional data file 2 contains example of a histogram of the 'fitness scores' of unique BLAT matches. Additional data file 3 further explains consensus splice site analyses and shows the squared values of probabilities computed. Additional data file 4 contains a file that can be uploaded to University of California at Santa Cruz Genome Brower to view all RACE products.

## Supplementary Material

Additional data file 1 Shown are examples of RACE PCR products on an agarose gel. Click here for file

Additional data file 2 The scores are computed using a subset (from one experiment) of our first set of RACE sequences, as described in the first row of Table 1. We then determined a reasonable threshold on these scores according to the overall distribution of the scores of those 'unique' matches (for every sequence, we choose the match with the highest score that locates on the expected chromosome, if one exists). We selected a threshold of 70 for the 'fitness scores', because this value clearly separates the two sets of matches, which we can interpret as high-quality and low-quality ones. Clearly, lowering the threshold will increase the sensitivity of our analysis, while decreasing the specificity. Click here for file

Additional data file 3 Further explanation of consensus splice site analyses : In order to decide the window size for the consensus splice site analysis, we considered a simplified model in which a nucleotide sequence of length *N *is generated by randomly selecting A, C, G, T with equal probability of 1/4, and then computed the probability (*prob_pattern*) of that sequence containing at least one pattern of a consensus splice site (for example, having either 'GT' or 'AG' in the sequence). This is as follows: *prob_pattern*(*N*) = *count_pattern*(*N*)/(4^*N*), where *count_pattern*(1) = 0, *count_pattern*(2) = 1, *count_pattern*(*N*) = 4^(*N *- 2) - *count_pattern*(*N *- 2) + 4 × *count_pattern*(*N *- 1), for *N *> 2. Although this formula does not take into account many sophisticated factors in reality, it can provide us a good guideline on selecting the window size for our analysis. This file shows the squared values of such probabilities (which can be considered as a lower bound of the probability for a random sequence to have a complete consensus pattern) for *N *ranging from 2 to 13. In the analysis of this paper, we selected the window size to be 8 to ensure at least twofold enrichment in the number of sequences that we identified compared with that in the simplified model, given the same number of sequences. Click here for file

Additional data file 4 This file can be uploaded to the University of California at Santa Cruz Genome Brower to view all RACE products. Click here for file

## References

[B1] ENCODE Project Consortium Identification and analysis of functional elements in 1% of the human genome by the ENCODE pilot project. Nature 2007 447 799 816 10.1038/nature05874 17571346 PMC2212820

[B2] Carninci P Kasukawa T Katayama S Gough J Frith MC Maeda N Oyama R Ravasi T Lenhard B Wells C The transcriptional landscape of the mammalian genome. Science 2005 309 1559 1563 10.1126/science.1112014 16141072

[B3] Kapranov P Cheng J Dike S Nix DA Duttagupta R Willingham AT Stadler PF Hertel J Hackermueller J Hofacker IL RNA maps reveal new RNA classes and a possible function for pervasive transcription. Science 2007 316 1484 1488 10.1126/science.1138341 17510325

[B4] ENCODE Project Consortium The ENCODE (ENCyclopedia Of DNA Elements) Project. Science 2004 306 636 640 10.1126/science.1105136 15499007

[B5] Kapranov P Willingham AT Gingeras TR Genome-wide transcription and the implications for genomic organization. Nat Rev Genet 2007 8 413 423 10.1038/nrg2083 17486121

[B6] Harrow J Denoeud F Frankish A Reymond A Chen CK Chrast J Lagarde J Gilbert JG Storey R Swarbreck D GENCODE: producing a reference annotation for ENCODE. Genome Biol 2006 7 Suppl 1 1 9 10.1186/gb-2006-7-s1-s4 PMC1810553 16925838

[B7] Gerhard DS Wagner L Feingold EA Shenmen CM Grouse LH Schuler G Klein SL Old S Rasooly R Good P MGC Project Team The status, quality, and expansion of the NIH full-length cDNA project: the Mammalian Gene Collection (MGC). Genome Res 2004 14 2121 2127 10.1101/gr.2596504 15489334 PMC528928

[B8] Wu JQ Garcia AM Hulyk S Sneed A Kowis C Yuan Y Steffen D McPherson JD Gunaratne PH Gibbs RA Large-scale RT-PCR recovery of full-length cDNA clones. Biotechniques 2004 36 690 696 15088387 10.2144/04364DD03

[B9] Wu JQ Shteynberg D Arumugam M Gibbs RA Brent MR Identification of rat genes by TWINSCAN gene prediction, RT-PCR, and direct sequencing. Genome Res 2004 14 665 671 10.1101/gr.1959604 15060008 PMC383311

[B10] Trinklein ND Karaoz U Wu J Halees A Force Aldred S Collins PJ Zheng D Zhang ZD Gerstein MB Snyder M Integrated analysis of experimental data sets reveals many novel promoters in 1% of the human genome. Genome Res 2007 17 720 731 10.1101/gr.5716607 17567992 PMC1891333

[B11] Denoeud F Kapranov P Ucla C Frankish A Castelo R Drenkow J Lagarde J Alioto T Manzano C Chrast J Prominent use of distal 5' transcription start sites and discovery of a large number of additional exons in ENCODE regions. Genome Res 2007 17 746 759 10.1101/gr.5660607 17567994 PMC1891335

[B12] Cooper SJ Trinklein ND Anton ED Nguyen L Myers RM Comprehensive analysis of transcriptional promoter structure and function in 1% of the human genome. Genome Res 2006 16 1 10 10.1101/gr.4222606 16344566 PMC1356123

[B13] Carninci P Sandelin A Lenhard B Katayama S Shimokawa K Ponjavic J Semple CA Taylor MS Engstrom PG Frith MC Genome-wide analysis of mammalian promoter architecture and evolution. Nat Genet 2006 38 626 635 10.1038/ng1789 16645617

[B14] Kim TH Barrera LO Qu C Van Calcar S Trinklein ND Cooper SJ Luna RM Glass CK Rosenfeld MG Myers RM Direct isolation and identification of promoters in the human genome. Genome Res 2005 15 830 839 10.1101/gr.3430605 15899964 PMC1142473

[B15] Bertone P Stolc V Royce TE Rozowsky JS Urban AE Zhu X Rinn JL Tongprasit W Samanta M Weissman S Global identification of human transcribed sequences with genome tiling arrays. Science 2004 306 2242 2246 10.1126/science.1103388 15539566

[B16] Cheng J Kapranov P Drenkow J Dike S Brubaker S Patel S Long J Stern D Tammana H Helt G Transcriptional maps of 10 human chromosomes at 5-nucleotide resolution. Science 2005 308 1149 1154 10.1126/science.1108625 15790807

[B17] Rinn JL Euskirchen G Bertone P Martone R Luscombe NM Hartman S Harrison PM Nelson FK Miller P Gerstein M The transcriptional activity of human chromosome 22. Genes Dev 2003 17 529 540 10.1101/gad.1055203 12600945 PMC195998

[B18] Rozowsky J Wu J Lian Z Nagalakshmi U Korbel JO Kapranov P Zheng D Dyke S Newburger P Miller P Novel transcribed regions in the human genome. Cold Spring Harb Symp Quant Biol 2006 71 111 116 10.1101/sqb.2006.71.054 17381286

[B19] Katayama S Tomaru Y Kasukawa T Waki K Nakanishi M Nakamura M Nishida H Yap CC Suzuki M Kawai J Antisense transcription in the mammalian transcriptome. Science 2005 309 1564 1566 10.1126/science.1112009 16141073

[B20] Rozowsky JS Newburger D Sayward F Wu J Jordan G Korbel JO Nagalakshmi U Yang J Zheng D Guigo R The DART classification of unannotated transcription within the ENCODE regions: associating transcription with known and novel loci. Genome Res 2007 17 732 745 10.1101/gr.5696007 17567993 PMC1891334

[B21] Kao HT Porton B Czernik AJ Feng J Yiu G Haring M Benfenati F Greengard P A third member of the synapsin gene family. Proc Natl Acad Sci USA 1998 95 4667 4672 10.1073/pnas.95.8.4667 9539796 PMC22548

[B22] Lachman HM Stopkova P Rafael MA Saito T Association of schizophrenia in African Americans to polymorphism in synapsin III gene. Psychiatr Genet 2005 15 127 132 10.1097/00041444-200506000-00009 15900227

[B23] Docherty AJ Lyons A Smith BJ Wright EM Stephens PE Harris TJ Murphy G Reynolds JJ Sequence of human tissue inhibitor of metalloproteinases and its identity to erythroid-potentiating activity. Nature 1985 318 66 69 10.1038/318066a0 3903517

[B24] SAGE Anatomic Viewer http://cgap.nci.nih.gov/SAGE/AnatomicViewer

[B25] Perocchi F Xu Z Clauder-Munster S Steinmetz LM Antisense artifacts in transcriptome microarray experiments are resolved by actinomycin D. Nucleic Acids Res 2007 35 e128 10.1093/nar/gkm683 17897965 PMC2095812

[B26] Kapranov P Drenkow J Cheng J Long J Helt G Dike S Gingeras TR Examples of the complex architecture of the human transcriptome revealed by RACE and high-density tiling arrays. Genome Res 2005 15 987 997 10.1101/gr.3455305 15998911 PMC1172043

[B27] Gish W States DJ Identification of protein coding regions by database similarity search. Nat Genet 1993 3 266 272 10.1038/ng0393-266 8485583

[B28] International Human Genome Sequencing Consortium Finishing the euchromatic sequence of the human genome. Nature 2004 431 931 945 10.1038/nature03001 15496913

[B29] Okazaki Y Furuno M Kasukawa T Adachi J Bono H Kondo S Nikaido I Osato N Saito R Suzuki H Analysis of the mouse transcriptome based on functional annotation of 60,770 full-length cDNAs. Nature 2002 420 563 573 10.1038/nature01266 12466851

[B30] Hastings ML Ingle HA Lazar MA Munroe SH Post-transcriptional regulation of thyroid hormone receptor expression by cis-acting sequences and a naturally occurring antisense RNA. J Biol Chem 2000 275 11507 11513 10.1074/jbc.275.15.11507 10753970

[B31] Li AW Murphy PR Expression of alternatively spliced FGF-2 antisense RNA transcripts in the central nervous system: regulation of FGF-2 mRNA translation. Mol Cell Endocrinol 2000 162 69 78 10.1016/S0303-7207(00)00209-4 10854699

[B32] Kelley RL Kuroda MI Noncoding RNA genes in dosage compensation and imprinting. Cell 2000 103 9 12 10.1016/S0092-8674(00)00099-4 11051542

[B33] Vanhee-Brossollet C Vaquero C Do natural antisense transcripts make sense in eukaryotes? Gene 1998 211 1 9 10.1016/S0378-1119(98)00093-6 9573333

[B34] Margulies M Egholm M Altman WE Attiya S Bader JS Bemben LA Berka J Braverman MS Chen YJ Chen Z Genome sequencing in microfabricated high-density picolitre reactors. Nature 2005 437 376 380 16056220 10.1038/nature03959 PMC1464427

[B35] Brenner S Johnson M Bridgham J Golda G Lloyd DH Johnson D Luo S McCurdy S Foy M Ewan M Gene expression analysis by massively parallel signature sequencing (MPSS) on microbead arrays. Nat Biotechnol 2000 18 630 634 10.1038/76469 10835600

[B36] Gromek K Kaczorowski T DNA sequencing by indexer walking. Clin Chem 2005 51 1612 1618 10.1373/clinchem.2004.046599 16037413

[B37] So AP Turner RF Haynes CA Increasing the efficiency of SAGE adaptor ligation by directed ligation chemistry. Nucleic Acids Res 2004 32 e96 10.1093/nar/gnh082 15247329 PMC484191

[B38] Bainbridge MN Warren RL Hirst M Romanuik T Zeng T Go A Delaney A Griffith M Hickenbotham M Magrini V Analysis of the prostate cancer cell line LNCaP transcriptome using a sequencing-by-synthesis approach. BMC Genomics 2006 7 246 10.1186/1471-2164-7-246 17010196 PMC1592491

[B39] Ross-Macdonald P Coelho PS Roemer T Agarwal S Kumar A Jansen R Cheung KH Sheehan A Symoniatis D Umansky L Large-scale analysis of the yeast genome by transposon tagging and gene disruption. Nature 1999 402 413 418 10.1038/46558 10586881

[B40] Coelho PS Kumar A Snyder M Genome-wide mutant collections: toolboxes for functional genomics. Curr Opin Microbiol 2000 3 309 315 10.1016/S1369-5274(00)00095-3 10851164

[B41] Mattick JS Makunin IV Non-coding RNA. Hum Mol Genet 2006 15 R17 R29 10.1093/hmg/ddl046 16651366

[B42] Prasanth KV Spector DL Eukaryotic regulatory RNAs: an answer to the 'genome complexity' conundrum. Genes Dev 2007 21 11 42 10.1101/gad.1484207 17210785

[B43] Zhu YY Machleder EM Chenchik A Li R Siebert PD Reverse transcriptase template switching: a SMART approach for full-length cDNA library construction. Biotechniques 2001 30 892 897 11314272 10.2144/01304pf02

[B44] Kent WJ BLAT: the BLAST-like alignment tool. Genome Res 2002 12 656 664 Article published online before March 2002 10.1101/gr.229202 11932250 PMC187518

[B45] Kent WJ Sugnet CW Furey TS Roskin KM Pringle TH Zahler AM Haussler D The human genome browser at UCSC. Genome Res 2002 12 996 1006 Article published online before print in May 2002 10.1101/gr.229102 12045153 PMC186604

[B46] Pruitt KD Tatusova T Maglott DR NCBI Reference Sequence (RefSeq): a curated non-redundant sequence database of genomes, transcripts and proteins. Nucleic Acids Res 2005 33 D501 D504 10.1093/nar/gki025 15608248 PMC539979

[B47] Universal ProbeLibrary Assay Design Center https://www.roche-applied-science.com/sis/rtpcr/upl/adc.jsp

