# Supplementary figures and images for: Systematic analysis of transcribed loci in ENCODE regions using RACE sequencing reveals extensive transcription in the human genome
Source: Genome Biol. 2008 Jan 3;9(1):R3. doi: 10.1186/gb-2008-9-1-r3 (PMC2395237; doi:10.1186/gb-2008-9-1-r3)

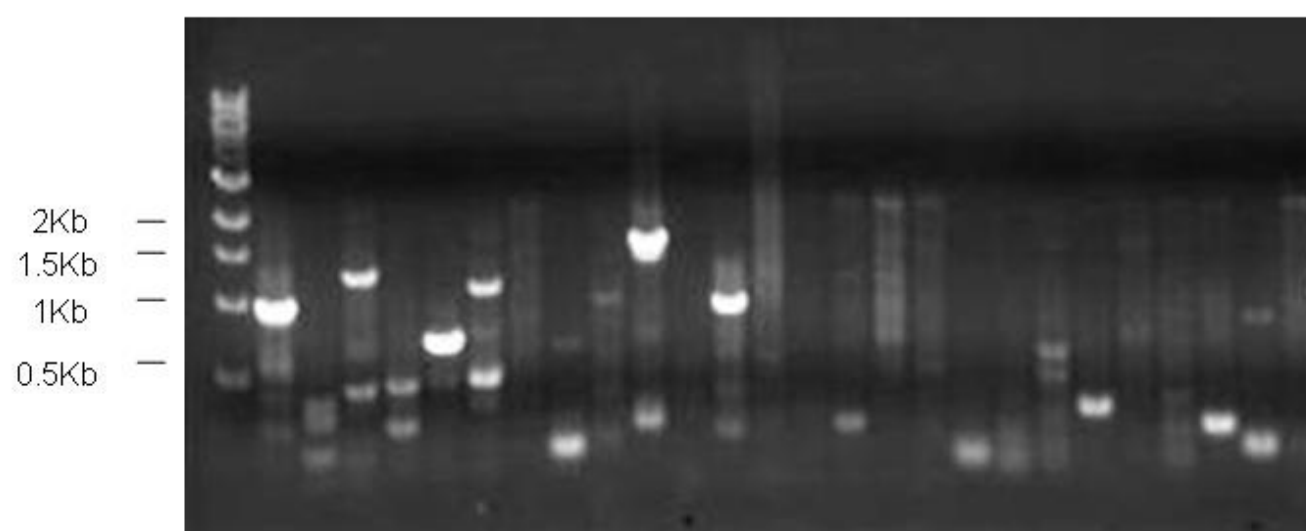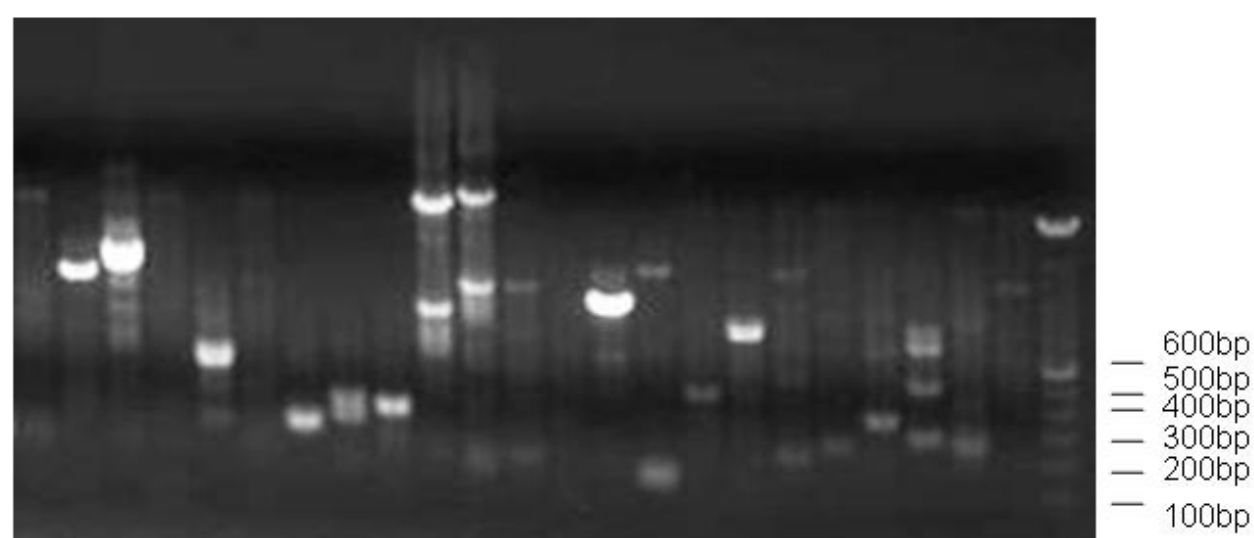

Supplement: Additional data file 1 — Shown are examples of RACE PCR products on an agarose gel. [file gb-2008-9-1-r3-S1.pdf]

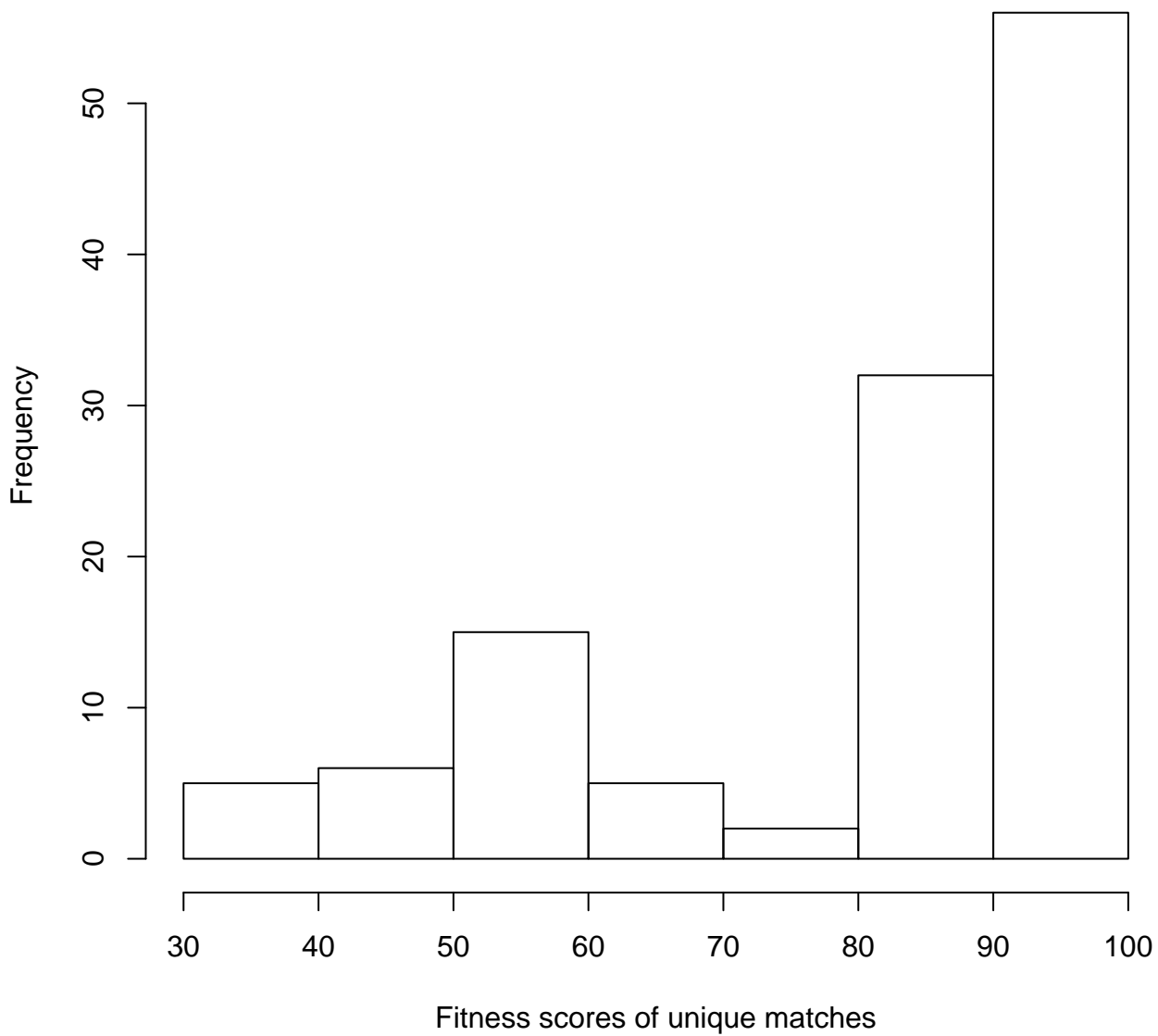

Supplement: Additional data file 2 — The scores are computed using a subset (from one experiment) of our first set of RACE sequences, as described in the first row of Table 1. We then determined a reasonable threshold on these scores according to the overall distribution of the scores of those 'unique' matches (for every sequence, we choose the match with the highest score that locates on the expected chromosome, if one exists). We selected a threshold of 70 for the 'fitness scores', because this value clearly separates the two sets of matches, which we can interpret as high-quality and low-quality ones. Clearly, lowering the threshold will increase the sensitivity of our analysis, while decreasing the specificity. [file gb-2008-9-1-r3-S2.pdf]

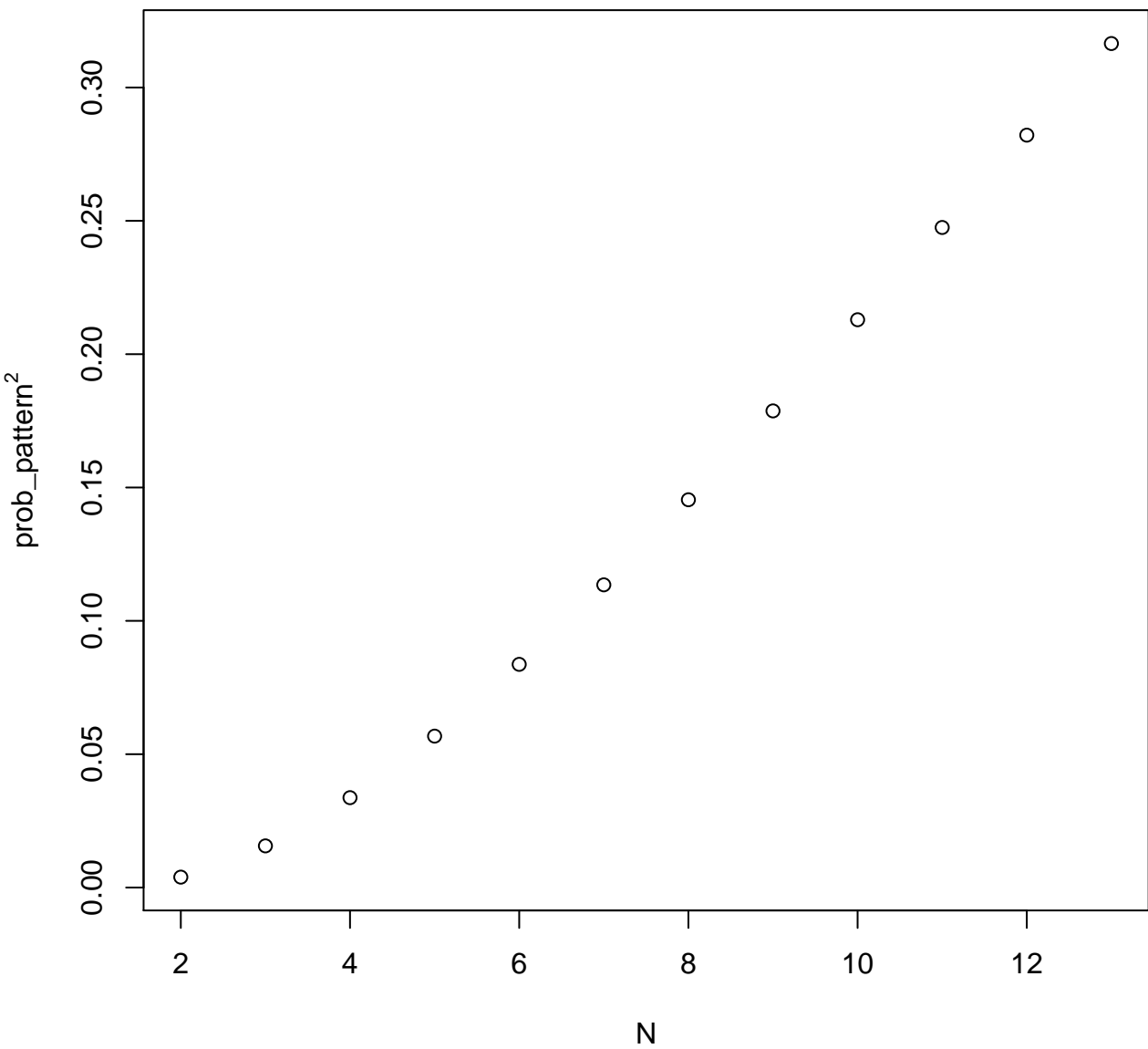

Supplement: Additional data file 3 — Further explanation of consensus splice site analyses : In order to decide the window size for the consensus splice site analysis, we considered a simplified model in which a nucleotide sequence of length N is generated by randomly selecting A, C, G, T with equal probability of 1/4, and then computed the probability (prob_pattern) of that sequence containing at least one pattern of a consensus splice site (for example, having either 'GT' or 'AG' in the sequence). This is as follows: prob_pattern(N) = count_pattern(N)/(4^N), where count_pattern(1) = 0, count_pattern(2) = 1, count_pattern(N) = 4^(N - 2) - count_pattern(N - 2) + 4 × count_pattern(N - 1), for N > 2. Although this formula does not take into account many sophisticated factors in reality, it can provide us a good guideline on selecting the window size for our analysis. This file shows the squared values of such probabilities (which can be considered as a lower bound of the probability for a random sequence to have a complete consensus pattern) for N ranging from 2 to 13. In the analysis of this paper, we selected the window size to be 8 to ensure at least twofold enrichment in the number of sequences that we identified compared with that in the simplified model, given the same number of sequences. [file gb-2008-9-1-r3-S3.pdf]
